# Supplementary material for: The Bronchoprotective Effects of Dual Pharmacology, Muscarinic Receptor Antagonist and β2 Adrenergic Receptor Agonist Navafenterol in Human Small Airways
Source: Cells. 2023 Jan 6;12(2):240. doi: 10.3390/cells12020240 (PMC9856842; doi:10.3390/cells12020240)
Supplement: Supplementary file 1 [file cells-12-00240-s001.zip › cells-2095894-supplementary.pdf]

Supplementary Figure

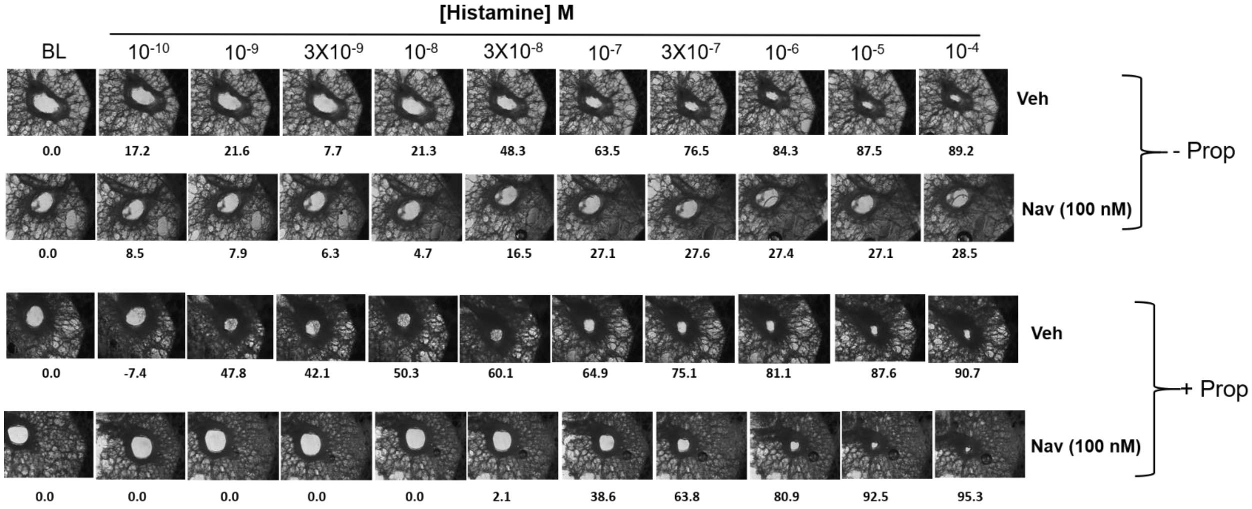

Figure S1. Representative airway images for histamine concentration response. 4 representative airways imaged after exposure to histamine (10^{-10} M to 10^{-4} M) in the presence of Navafenterol (Nav) 100 nM -/+ 10 uM propranolol (Prop). Following experimental exposures, the airway is treated with incremental concentrations of histamine (5 min) and imaged under a light microscope at 40X magnification. The images are analyzed by measuring the airway lumen area (white space) using Image J software. The % reduction in the lumen area in each individual airway is calculated by normalizing each luminal area to that airway's baseline (BL). Thus, the airway luminal area change at baseline condition in each airway will be 0.0 %. The % change in airway luminal area in response to contractile agonist histamine is called "% bronchoconstriction" (shown under each image). Because the luminal area change at each concentration of histamine is normalized to the baseline of the same airway, variations in actual size and shape of the airways used in different experimental groups are eliminated from the analysis. (Images represent, for each condition, 1 technical replicate from a single donor; Veh – 0.1% DMSO).
